# Draft genome sequences of human adenovirus F associated with acute gastroenteritis in Blantyre, Malawi

**DOI:** 10.1128/mra.00545-25

**Published:** 2025-10-21

**Authors:** Flywell Kawonga, Ernest Matambo, End Chinyama, Chimwemwe Mhango, Clara Majengo, Josephine Msowoya, Benjamin Kumwenda, Celeste M. Donato, Arox W. Kamng'ona, Milton T. Mogotsi, Nkosazana Shange, Ayodeji E. Ogunbayo, Francis E. Dennis, Martin M. Nyaga, Chrispin Chaguza, Khuzwayo C. Jere

**Affiliations:** 1 Malawi-Liverpool-Wellcome Programme, Queen Elizabeth Central Hospital 299619, Blantyre, Malawi; 2 Institute of Infection, Veterinary & Ecological Sciences, Faculty of Health and Life Sciences, University of Liverpool 105724, Liverpool, United Kingdom; 3 Department of Medical Laboratory Sciences, Faculty of Biomedical Sciences and Health Profession, Kamuzu University of Health Sciences 37610, Blantyre, Malawi; 4 Department of Pharmacy, School of Life Sciences and Allied Health Professions, Kamuzu University of Health Sciences 838627, Blantyre, Malawi; 5 Department of Pathology, School of Medicine and Oral Health, Kamuzu University of Health Sciences 37610, Blantyre, Malawi; 6 Biomedical Sciences Department, School of Life Sciences and Allied Health Professions, Kamuzu University of Health Sciences 838627, Blantyre, Malawi; 7 Enteric Diseases Group, Murdoch Children's Research Institute 34361, Parkville, Australia; 8 Next Generation Sequencing Unit, School of Biomedical Sciences and Division of Virology, Faculty of Health Sciences, University of Free State 108140 , Bloemfontein, South Africa; 9 Department of Electron Microscopy and Histopathology, Noguchi Memorial Institute for Medical Research, University of Ghana 118922, Accra, Ghana; 10 Department of Host-Microbe Interactions, St Jude Children’s Research Hospital 5417, Memphis, Tennessee, USA; Katholieke Universiteit Leuven, Leuven, Belgium

**Keywords:** human adenovirus F (HAdV-F), genotype 40/41, pediatric gastroenteritis, whole-genome sequencing, Malawi, phylogenetics, adenovirus genomics, SACEV, enteric viruses

## Abstract

Human adenovirus F (HAdV-F), genotype 40/41, ranks as the second leading cause of pediatric viral gastroenteritis globally. Here, we report four draft genomes of HAdV-F from Malawi, obtained from children with acute gastroenteritis at Queen Elizabeth Central Hospital and Bangwe Health Centre, Blantyre, between 2012 and 2024.

## ANNOUNCEMENT

Human adenovirus F (HAdV-F), genotypes 40 and 41 are major causes of pediatric viral gastroenteritis, accounting for 2.8–11.8% of diarrheal cases in infants worldwide (1). The HAdV belongs to the *Mastadenovirus faecale* genus within the family *Adenoviridae*. The average genome sizes of HAdV-F40 and HAdV-F41 are 34,214 and 34,188 base pairs, respectively (2, 3). In Malawi, HAdV-F (genotype 40/41) was the second most frequently detected pathogen associated with diarrhea, identified in 29.1% of cases at Queen Elizabeth Central Hospital (QECH) in our previous study (4). No genomic studies of HAdV-F have been conducted in Malawi. This report presents four draft genome sequences of HAdV-F from children under 5 years old with acute gastroenteritis at QECH and Bangwe Health Centre, as part of the Sequencing and Antigenic Cartography of Enteric Viruses (SACEV) project.

Ten samples per month were screened by polymerase chain reaction (PCR) using customized TaqMan Array Cards as previously described (5). Four positive adenovirus samples with PCR cycle threshold (Ct) <35 were selected for whole genome sequencing. Nucleic acid was extracted from adenovirus-positive stool samples using QIAamp Fast DNA Stool mini kit (Qiagen, Hilden, Germany). DNA extracts were quantified using a High Sensitivity dsDNA Assay on a Qubit Flex Fluorometer (Thermo Fisher Scientific, USA).

The HAdV-F described here was generated by shotgun metagenomic sequencing. Extracted DNA was subjected to whole transcriptome amplification using a Qiagen FX Whole Transcriptome Amplification kit, generating genomic sequences of all the genetic material present in the sample. Genomic libraries were prepared using the Illumina DNA Prep kit (Illumina, USA) before being sequenced on the Illumina NextSeq 2000 platform using a P1 flow cell and 300-cycle reagent kit (2 × 150  bp paired-end reads). Quality control was performed using FastQC 0.11.7 (https://www.bioinformatics.babraham.ac.uk/projects/fastqc/).

Low-quality reads were trimmed using Trimmomatic (v0.39) (6). To remove human reads, the trimmed sequences were aligned to the human genome (Homo_sapiens.GRCh38.dna.primary_assembly.fa, NCBI accession ID: GCA_000001405.15) using Bowtie2 2.5.4 with default parameters (7). The non-human reads were mapped to NC_001454.1 reference genome using the Burrows-Wheeler Alignment 0.7.18-r1243-dirty with default parameters (8). Consensus genomes were generated by calling high-confidence bases from aligned sequence reads using iVar (v1.4.4) with default parameters (9). The whole-genome assemblies were further annotated using Prokka 1.14.6 (10). The genome assemblies are considered drafts due to unconfirmed 5′ and 3′ terminal sequences, although all major coding regions were successfully recovered. BLASTn of the assembled genomes showed that BID128S1 had 99.89% nucleotide similarity to MK962807.1, whereas BID1NKS2, CQA14XS1, and CQA185S1 had 99.77%, 99.71% and 99.76% similarity to NC_001454.1, respectively. The depth of the genome assemblies was checked using samtools 1.21 (https://www.htslib.org/). The reads and assembly characteristics are summarized in Table 1.

**TABLE 1:** Summary of the assembly details and statistics

	BID128S1	BID1NKS2	CQA14XS1	CQA185S1
Collection year	2012	2013	2023	2024
Sample type	Stool	Stool	Stool	Stool
Cycle threshold (Ct)	23.8	9.8	16.9	27
Sequencing platform	NextSeq 2000	NextSeq 2000	NextSeq 2000	NextSeq 2000
Total number of reads generated	507,336	515,370	536,530	654,632
Total mapped reads to the reference	296,038	267,408	93,055	529,873
Raw sequence length	36–151	36–151	36–151	36–151
Genome length	34,013	34,207	34,204	34,204
GC (%)	50	51	52	49
Genome depth	972.186×	1027.25×	335.299×	1951.61×
Reference nucleotide identity (%)	99.8	99.7	99.7%	99.7%
CDS	35	30	31	31
Genotype	HAdV-F41	HAdV-F40	HAdV-F40	HAdV-F40
GenBank accession number	PV637361	PV612151	PV637362	PV637360

**Fig 1:**
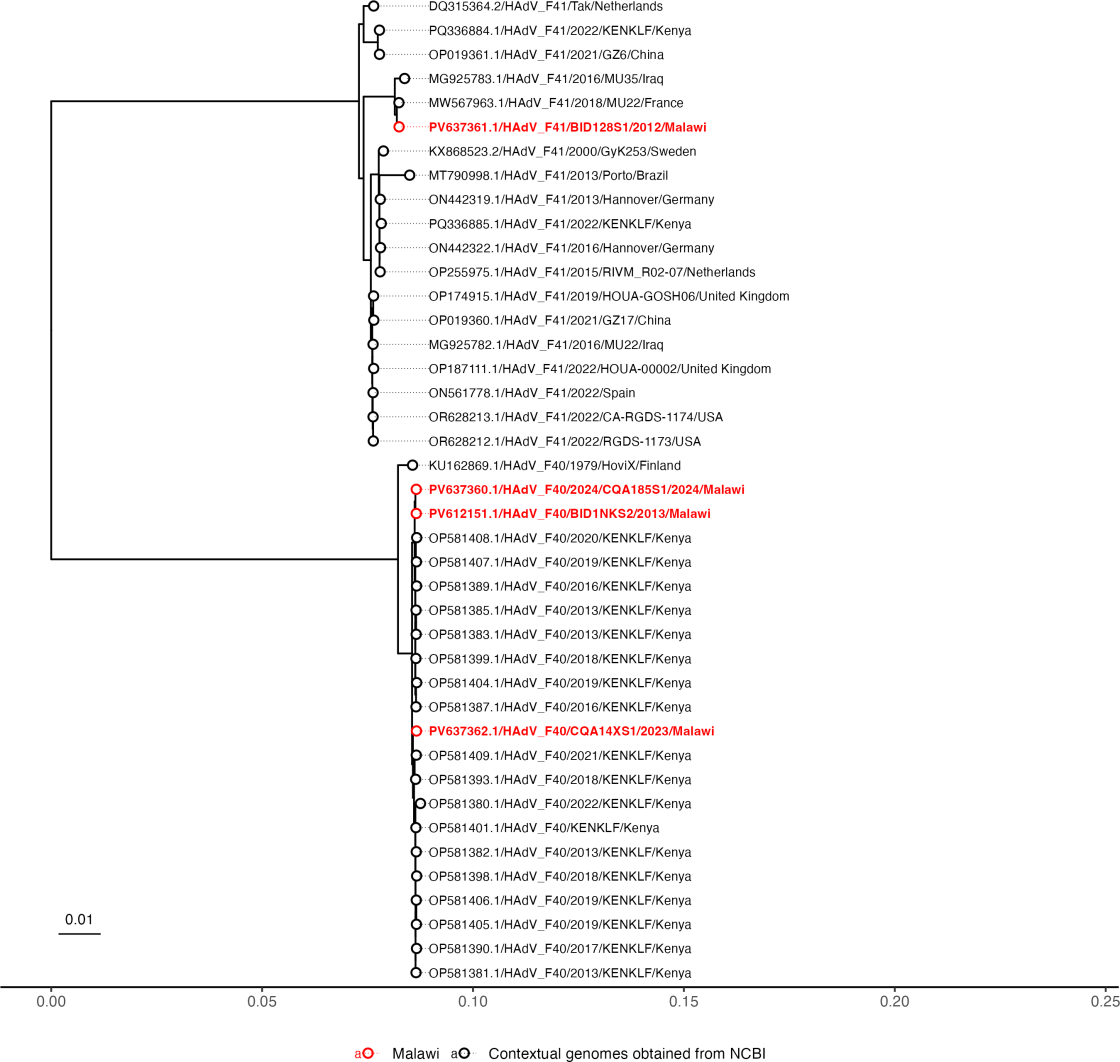
Contextual genomes of HAdV-40 and HAdV-41 were retrieved from the NCBI Virus database, selected based on complete coding sequences and broad global representation. Multiple sequence alignment was performed using MAFFT v7 with default parameters, and a maximum likelihood phylogenetic tree was inferred using IQ-TREE V2.4.0 with the GTR + G substitution model. Branch support was assessed using 1,000 ultrafast bootstrap replicates. The resulting tree was imported into R using the read.tree() function from the ape package (11) and rooted at the *Psittacine aviadenovirus C* (NCBI Accession: NC_075452.1) using the root() function to establish evolutionary directionality. For clarity, the outgroup was removed using the drop.tip() function from the same package. Strain metadata are used to annotate phylogenetic tree tips, distinguishing Malawian sequences from contextual genomes from NCBI. Malawian strains are shown in red, and contextual genomes are shown as black circles. The tips display the accession numbers, strain names, year of detection, and country of detection.

The assembled genomes were identified as human mastadenovirus F using the Genome Detective Virus Tool (https://doi.org/10.1093/bioinformatics/bty695). Genotyping identified BID1NKS2, CQA14XS1, and CQA185S1 as HAdV-F40, and BID128S1 as HAdV-F41, based on the highest sequence similarity from BLASTn analysis. Figure 1 shows the phylogeny of Malawian sequences alongside global contextual genomes, with the Malawian genomes clustering closely with sequences from Kenya and France.

## Data Availability

The whole-genome assemblies for samples BID128S1, BID1NKS2, CQA14XS1, and CQA185S1 have been deposited in GenBank under accession numbers PV637361, PV612151, PV637362, and PV637360, respectively. The BioProject accession number for the Sequence Read Archive (SRA) is PRJNA1276427.

## References

[1] Chandra P, Lo M, Mitra S, Banerjee A, Saha P, Okamoto K, Deb AK, Ghosh SK, Manna A, Dutta S, Chawla-Sarkar M. 2021. Genetic characterization and phylogenetic variations of human adenovirus-F strains circulating in eastern India during 2017-2020. J Med Virol 93:6180–6190. doi:10.1002/jmv.27136 34138479

[2] Lemiale F, Haddada H, Nabel GJ, Brough DE, King CR, Gall JGD. 2007. Novel adenovirus vaccine vectors based on the enteric-tropic serotype 41. Vaccine (Auckl) 25:2074–2084. doi:10.1016/j.vaccine.2006.11.025 PMC2584667 17250935

[3] van Loon AE, Ligtenberg M, Reemst AM, Sussenbach JS, Rozijn TH. 1987. Structure and organization of the left-terminal DNA regions of fastidious adenovirus types 40 and 41. Gene 58:109–126. doi:10.1016/0378-1119(87)90034-5 2961652

[4] Iturriza-Gómara M, Jere KC, Hungerford D, Bar-Zeev N, Shioda K, Kanjerwa O, Houpt ER, Operario DJ, Wachepa R, Pollock L, Bennett A, Pitzer VE, Cunliffe NA. 2019. Etiology of diarrhea among hospitalized children in Blantyre, Malawi, following rotavirus vaccine introduction: a case-control study. J Infect Dis 220:213–218. doi:10.1093/infdis/jiz084 30816414 PMC6581894

[5] Liu J, Gratz J, Amour C, Nshama R, Walongo T, Maro A, Mduma E, Platts-Mills J, Boisen N, Nataro J, Haverstick DM, Kabir F, Lertsethtakarn P, Silapong S, Jeamwattanalert P, Bodhidatta L, Mason C, Begum S, Haque R, Praharaj I, Kang G, Houpt ER. 2016. Optimization of quantitative PCR methods for enteropathogen detection. PLoS One 11:e0158199. doi:10.1371/journal.pone.0158199 27336160 PMC4918952

[6] Bolger AM, Lohse M, Usadel B. 2014. Trimmomatic: a flexible trimmer for Illumina sequence data. Bioinformatics 30:2114–2120. doi:10.1093/bioinformatics/btu170 24695404 PMC4103590

[7] Langmead B, Salzberg SL. 2012. Fast gapped-read alignment with Bowtie 2. Nat Methods 9:357–359. doi:10.1038/nmeth.1923 22388286 PMC3322381

[8] Li H, Durbin R. 2009. Fast and accurate short read alignment with Burrows-Wheeler transform. Bioinformatics 25:1754–1760. doi:10.1093/bioinformatics/btp324 19451168 PMC2705234

[9] Grubaugh ND, Gangavarapu K, Quick J, Matteson NL, De Jesus JG, Main BJ, Tan AL, Paul LM, Brackney DE, Grewal S, Gurfield N, Van Rompay KKA, Isern S, Michael SF, Coffey LL, Loman NJ, Andersen KG. 2019. An amplicon-based sequencing framework for accurately measuring intrahost virus diversity using PrimalSeq and iVar. Genome Biol 20:8. doi:10.1186/s13059-018-1618-7 30621750 PMC6325816

[10] Seemann T. 2014. Prokka: rapid prokaryotic genome annotation. Bioinformatics 30:2068–2069. doi:10.1093/bioinformatics/btu153 24642063

[11] Paradis E, Schliep K. 2019. Ape 5.0: an environment for modern phylogenetics and evolutionary analyses in R. Bioinformatics 35:526–528. doi:10.1093/bioinformatics/bty633 30016406

